# Applying amplification refractory mutation system technique to detecting cell-free fetal DNA for single-gene disorders purpose

**DOI:** 10.3389/fgene.2023.1071406

**Published:** 2023-04-11

**Authors:** Yu Tan, Hui Jian, Ranran Zhang, Jing Wang, Cong Zhou, Yuanyuan Xiao, Weibo Liang, Li Wang

**Affiliations:** ^1^ Department of Medical Genetics, West China Second University Hospital, Sichuan University, Chengdu, Sichuan, China; ^2^ Key Laboratory of Birth Defects and Related Diseases of Women and Children (Sichuan University), Ministry of Education, Chengdu, Sichuan, China; ^3^ Department of Laboratory Medicine, West China Second University Hospital, Sichuan University, Chengdu, Sichuan, China; ^4^ Department of Forensic Genetics, West China School of Basic Medical Sciences and Forensic Medicine, Sichuan University, Chengdu, Sichuan, China

**Keywords:** non-invasive prenatal diagnosis (NIPD) for single-gene disorders, amplification refractory mutation system (ARMS), cell-free fetal DNA (cffDNA), allele-specific primers, capillary electrophoresis

## Abstract

Non-invasive prenatal diagnosis for single-gene disorders (NIPD) is still in development and deserves further study. The advent of next-generation sequencing technology significantly improved the detection of multiple mutations for non-invasive prenatal diagnosis for single-gene disorder purposes. However, bespoke amplicon-based NGS assays are costly. In this study, we developed a new strategy for non-invasive prenatal screening for single-gene disorders based on a capillary electrophoresis (CE) platform using an amplification refractory mutation system (ARMS)-PCR technique. Allele-specific primers for several disease-correlated mutations were designed, and subsequently, sensitivity and specificity assays were conducted. Assays on simulated two-person DNA mixtures showed that three primers targeting the mutant allele could detect minor DNA components in 1:500 mixtures. All primers showed positive results at 0.01 ng of the template DNA. Cell-free fetal DNA was extracted from a pregnant woman’s peripheral blood for the detection of paternally inherited mutations. Our results showed that one primer successfully amplified the mutant allele of fetal DNA in maternal plasma, which was confirmed by genotyping the genomic DNA extracted from amniotic fluid. This study suggested that the ARMS-PCR technique, a fast and cost-effective method, might be a promising method used to target *de novo* or paternally inherited pathogenic mutations in maternal plasma.

## 1 Introduction

The discovery of cell-free fetal DNA (cffDNA) in maternal plasma was first reported in 1997 (Lo et al., 1997). Since then, non-invasive prenatal testing (NIPT) for aneuploidy has been developed rapidly, and now, this technique is routinely applied in clinical practice for trisomy 13, 18, and 21. However, the development of non-invasive prenatal diagnosis for single-gene disorders has been relatively slower than that of NIPT. This may be because NIPD represents a smaller market opportunity, and many cases require a bespoke, patient-specific, or disease-specific testing strategy. Therefore, the complexity of detection and the lack of commercial drive might account for the slow development of this technique.

The first NIPD testing approved for use in clinical settings was in 2011 for fetal sex determination (Hill et al., 2011; Clausen, 2014; Jenkins et al., 2018) and now is routinely used for patients with a family history of X-linked disorders, such as Duchenne muscular dystrophy (Xu et al., 2015). The detection method was mainly based on haplotype-based analysis. This approach required the grouping of single-nucleotide polymorphisms (SNPs) into different informative categories based on parents’ genotypes. Some researchers also reported that this method could also be applied to the diagnosis of autosomal recessive disorders like GJB2-associated hearing impairment (Xiong et al., 2015). The analysis of fetal DNA in maternal plasma based on the aforementioned haplotype analysis was called the relative haplotype dosage (RHDO). The RHDO method was successfully used to test cystic fibrosis (CF) and congenital adrenal hyperplasia (CAH) (Drury et al., 2016).

Another method applied to NIPD was droplet digital PCR (ddPCR). The principle of ddPCR is that the template DNA is partitioned into tens of thousands of droplets, and each droplet contains one or zero copies of the target gene. Therefore, the concentration of the target gene can be obtained by calculating the proportion of positive droplets. Because of this, ddPCR was applied to NIPD for the detection of paternal or *de novo* disease-causing mutations including achondroplasia (FGFR3) and neonatal diabetes (KCNJ11) (Orhant et al., 2016; Perlado et al., 2016; De Franco et al., 2017).

Next-generation sequencing is developing rapidly, and this technology is also being applied to NIPD. For example, target next-generation sequencing has been reported for the detection of skeletal dysplasia (Dan et al., 2016). In addition, next-generation sequencing panels covering multiple target genes or mutations make it possible to detect several mutations simultaneously (Chitty et al., 2015).

**FIGURE 1:**
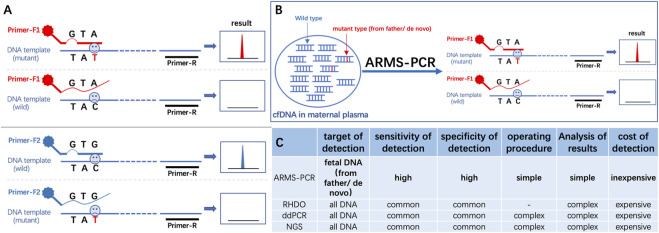
**(A)** Schematic diagram of the ARMS-PCR primer design principle. **(B)** Schematic diagram of ARMS-PCR for detection of fetal free DNA in maternal plasma. **(C)** Comparison of ARMS-PCR with the RHDO, ddPCR, and NGS methods.

In this study, we developed a new strategy to detect paternal or *de novo* mutations from maternal plasma for screening of single-gene disorders of the fetus. The new strategy is mainly based on the amplification refractory mutation system (ARMS)-PCR technique. The ARMS-based PCR technique is a classical and well-developed method for SNP genotyping (Muñoz et al., 2009). By designing two forward allele-specific primers targeting two different allele sequences of a SNP, respectively, this technique can successfully genotype a SNP (Figure 1A). Moreover, our previous studies have shown that allele-specific primers can target specific alleles (e.g., allele A) from a high background of other alleles (e.g., allele T/C/G) (Tan et al., 2018). In addition, circulating cell-free DNA (cfDNA) in maternal plasma is a mixture of predominant maternal DNA derived from the hematopoietic system of the mother (Scotchman et al., 2020) and fetal DNA released from apoptosis of cytotrophoblast cells (Lo et al., 2010). cffDNA accounts for about 5%–20% of total cfDNA in maternal blood, increasing throughout pregnancy (Scotchman et al., 2020). These features indicate that if the fetus carries a *de novo* mutation or a paternal mutation, it can theoretically be detected in the peripheral blood of a pregnant woman by allele-specific primers (Figure 1B). In this study, we conducted an assay to detect paternal mutations from the plasma of a pregnant woman through the ARMS-PCR technique. The couple in this study had single-gene carrier screening before pregnancy, and the screening results indicated that the father carried four disease-associated mutations which could be passed on to the fetus. The mother was a wild type of these four variants. Therefore, we designed allele-specific primers for these mutant alleles based on the ARMS-PCR technique and amplified the cell-free DNA extracted from the maternal plasma. Results of this study suggested that the ARMS-PCR technique could be used to detect cffDNA and may be a promising technology for detecting *de novo* and paternally inherited disease-causing mutations. Compared with the RHDO, ddPCR, and NGS methods, the ARMS-PCR technique combined with the CE platform is fast, economical, and easy to handle (Figure 1C).

## 2 Materials and methods

### 2.1 Sample collection and DNA preparation

A measure of 2 mL peripheral venous blood was collected from a couple; the wife was less than 28 weeks pregnant. Ethylenediaminetetraacetic acid (EDTA) was added to the collection tube for anticoagulation. Meanwhile, an additional 10 mL of maternal peripheral venous blood was collected for plasma separation. In addition, 2 mL of the amniotic fluid sample was collected from the pregnant woman for further validation studies. The current study was approved by the Medical Ethics Committee of the West China Second University Hospital of Sichuan University (Chengdu, China), and written informed consent was obtained from the patients.

The genomic DNA was extracted using a whole blood extraction kit (BioTeke, Beijing, China). Maternal plasma was separated through a two-step centrifugation process. In the first step, 10 mL peripheral venous blood was centrifuged at 1,600 g for 10 min at 4°C to obtain the upper-layer plasma. Then, second centrifugation at 16,000 g for 10 min at 4°C was performed for the recovered plasma in the first step. The cell-free circulating DNA was extracted from 1 mL of the plasma using the QIAamp Circulating Nucleic Acid Kit (QIAGEN AG, Basel, Switzerland) according to the manufacturer’s protocol. Fetal DNA from amniotic fluid samples was extracted through the DNeasy Blood and Tissue Kit (QIAGEN, Germany). Both the genomic and circulating DNA samples were stored at −20°C.

### 2.2 Clinical information on the involved couple and target mutation screening

The volunteer couple in this study underwent single-gene carrier screening before pregnancy. The screening result showed that the wife carried one pathogenic mutation, i.e., c.109G>A of *GJB2* gene (Abe et al., 2000), and the husband carried four disease-associated mutations, i.e., GJB2 c.235del (Fuse et al., 1999), DYSF c.4585C>T (Guglieri et al., 2008), SLC26A4 c.2236-25T>A (Yuan et al., 2012), and PAH c.158G>A (Park et al., 1998). Detailed information is shown in Table 1. If their fetus inherited both variants of the *GJB2* gene from the parents, it may be born with a hearing impairment. For further clarity,, prenatal diagnoses were carried out by sequencing amniotic fluid samples. In our study, the four mutations carried by the father (GJB2 c.235del, DYSF c.4585C>T, SLC26A4 c.2236-25T>A, and PAH c.158G>A) were selected as our candidate loci. We conducted an assay to detect these four mutations of the fetal DNA in the maternal plasma and compared the results with those of amniocentesis.

**TABLE 1:** Information on four SNVs.

Number	Gene	Mutation	Chromosome location (hg19/GRCh37)	Mother	Father
No. 1	*GJB2*	c.235delG	chr13:20763486	Wild type	c.235delG, heterozygote
No. 2	*DYSF*	c.4585C>T	chr2:71883367	Wild type	c.4585C>T, heterozygote
No. 3	*SLC26A4*	c.2236-25T>A	chr7:107352959	Wild type	c.2236-25T>A, heterozygote
No. 4	*PAH*	c.158G>A	chr12:103306579	Wild type	c.158G>A, heterozygote

### 2.3 Genotyping

#### 2.3.1 Primer design

For each candidate variant in this study, we designed three primers to detect both alleles of the variants. A common reverse primer (R primer) was located downstream of the variant, and two allele-specific forward primers (F-A and F-B primers) were designed with their 3′ ends complementary to the wild-type allele and mutant allele, respectively. To enhance the specificity of primers, a deliberate mismatch was introduced at the antepenultimate or penultimate base at the 3′ ends of the primers targeting the mutant allele. For mutations involving deletions, F-A primers were exactly complementary to the wild-type sequences, while F-B primers lacked the deletion sequences at the 3’ end and exceeded the deletion point by 2–6 nucleotides. Primer sequences are shown in Supplementary Table S1.

#### 2.3.2 PCR conditions and genotyping

PCR amplifications were performed in 10-μL reactions containing 5 μL Multiplex PCR Mix (QIAGEN, Hilden, Germany), 0.5 μL forward primer and reverse primer mix (3 μM each), 3.5 μL nuclease-free water, and 1 μL genomic DNA (1 ng/μL). Thermocycling was performed in the Mastercycler^®^ Nexus Gradient instrument (Eppendorf, Hamburg, Germany) under the following conditions: initial denaturation at 95°C for 15 min; 31 cycles of 30 s at 94°C, 90 s at 58°C, and 60 s at 72°C; followed by a final extension step of 30 min at 60°C.

PCR products were separated and detected by capillary electrophoresis using the 3130XL Genetic Analyzer (Applied Biosystems, Foster City, CA, United States) by adding 1 μL of the product to 9 μL mixture of AGCU marker 500 (AGCU ScienTech Incorporation, Wuxi, China). Electrophoretic conditions were 9 s at 3 kV for injection and 1,000 s at 15 kV for the run. The data were analyzed using GeneMapper ID-X v1.2 software (Thermo Fisher Scientific, MA, United States), with the peak height threshold set at 50 RFU for each fluorescent dye.

### 2.4 Sensitivity and specificity of primers

Sensitivity assays were conducted to decide the detection threshold of each primer. Therefore, DNA samples with heterozygous alleles were selected to assess the minimum amount of template required for each allele-specific primer. DNA samples diluted to 1, 0.5, 0.2, 0.1, 0.05, 0.025, 0.01, 0.005, 0.0025, and 0.001 ng/μL were amplified according to the PCR conditions described in Section 3.2.

Since F-B primers are designed to target mutant sequences of the fetus in maternal plasma, the specificity and sensitivity of the primers are very important. For this purpose, we used different ratios (1:50, 1:100, 1:200, and 1:500) of two-person DNA mixtures to simulate the state of cell-free DNA in maternal plasma. Then, F-B primers were used to amplify the alleles of the minor components in mixtures to judge the ability of the primers to be used for the detection of the cell-free fetal DNA. For all simulated mixtures, the major contributor was a wild type for a given locus, while the minor contributor was heterozygous of the mutant allele.

### 2.5 Non-invasive prenatal testing

To explore if the ARMS-PCR technique can be used for non-invasive prenatal screening for single-gene disorders, four variants of the father were selected as our target loci. We used F-B primers of the four mutations to amplify cell-free DNA extracted from the maternal plasma to determine whether the fetus inherited these mutants from the father. The genomic DNA from the amniotic fluid was also sequenced at these four loci for double confirmation.

## 3 Results

### 3.1 PCR sensitivity

Sensitivity was evaluated for eight allele-specific primers of the four variants. The results showed that three primers obtained positive results at 0.01 ng of the template DNA. Another three of the primers’ thresholds were 0.005 ng. One primer reached 0.025 ng, and the remaining primer even reached 0.0025 ng. However, the sensitivity of F-B primers which annealed to the mutant sequences was generally lower than that of F-A primers. The results are shown in Supplementary Figure S1. Two of the F-B primers only showed positive results at 0.01 ng.

### 3.2 Simulated mixture analysis

**FIGURE 2:**
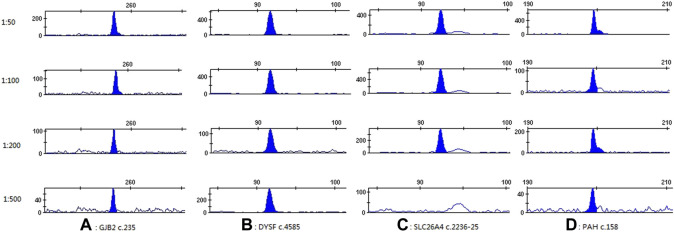
Specificity results of primers targeting the mutant allele. **(A–D)** indicate GJB2 c.235, DYSF c.4585, SLC26A4 c.2236-25, and PAH c.158, respectively.

Because F-B primers were designed to target the mutant allele, the specificity of F-B primers is very important to ensure an accurate genotype. Homemade two-person DNA mixtures were used to simulate a mixture of cell-free fetal DNA and maternal DNA in the mother’s peripheral blood plasma. Of the four allele-specific primers, three could successfully amplify minor component alleles from a 1:500 mixture without being affected by the major component alleles, while the other primer only achieved a ratio of 1:200. Detail information is shown in Figure 2.

### 3.3 Non-invasive prenatal testing

**FIGURE 3:**
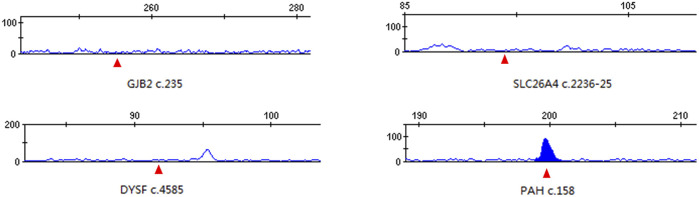
Detection results of cffDNA from maternal plasma. The red triangle indicates the location of the expected peak of each mutant allele.

The concentration of the extracted cell-free DNA was 0.406 ng/μL. Because the sensitivity of F-B primers varied, different amounts of the template DNA were added to the PCR reactions to meet the minimum amount of DNA required for each primer. As shown in Figure 3, negative, negative, negative, and positive results were obtained for GJB2 c.235del, DYSF c.4585C>T, SLC26A4 c.2236-25T>A, and PAH c.158G>A, respectively, when the cell-free DNA extracted from the maternal plasma was amplified with F-B primers.

To verify the accuracy of the aforementioned ARMS-PCR results, F-B primers were also used to amplify genomic DNA extracted from the amniotic fluid. Results showed that the fetus was wild-type, wild-type, mutant, and mutant for GJB2 c.235, DYSF c.4585, SLC26A4 c.2236-25, and PAH c.158, respectively. Sanger sequencing for the aforementioned four SNVs was also conducted for double confirmation (Supplementary Figure S2). The sequencing results were consistent with the results of the amniotic fluid genomic DNA amplified by F-B primers.

## 4 Discussion

NIPD for monogenic disorders is an emerging technique, and further research is still needed. First, the detection of single-gene disorders from maternal plasma is much more difficult than aneuploidy screening. Although several methods have been developed for NIPD, the accuracy and reproducibility of these methods require more experimental evaluation. Second, the cost of detection is also a concern of whether the technology can be widely used. In this study, we applied the ARMS-PCR technique to detect paternally inherited mutation from the cell-free DNA extracted from the maternal plasma. Detection can be easily achieved by designing a specific primer that only anneals to the mutant sequence. For the four mutations detected in our study, amniotic fluid DNA sequencing results demonstrated that the fetus only inherited two of them. However, ARMS-PCR results were consistent with amniotic fluid DNA sequencing at only three loci, including the detection of one mutant allele (PAH c.158G>A). The detection of another failed mutation (SLC26A4 c.2236-25T>A) might be because the primer was not sensitive enough. Therefore, in our later study, we will focus on promoting the sensitivity of primers by altering reaction parameters or increasing PCR cycling numbers. Although there might be false negative results using the ARMS-PCR technique, the successful detection of one mutant allele in this study at least proved that this method is helpful and might be a promising technique for the detection of mutations of the fetal DNA.

A key point affecting detection based on the ARMS-PCR technique is the primer specificity, especially the specificity of the primer targeting the mutant allele. The results of simulated mixture assays showed that three of the four primers reached 1:500, while the remaining primer only reached 1:200. For those primers with lower specificity, non-specific product peaks or peaks of the wild-type allele will appear in the electrophoretogram, which will interfere with the interpretation of the results or lead to false positive results. Previous reports on the ARMS-PCR technique indicated that an additional deliberate mismatch introduced at the antepenultimate base of the 3’ end of primers was superior to that at the penultimate base (Liu et al., 2012). In addition, some PCR additives, such as betaine, have been reported to help reduce non-specific amplification and improve the stringency of PCR reactions (Lajin et al., 2013). Therefore, we will consider these methods to promote the specificity of the primers in our future studies.

Another factor we should consider when designing allele-specific primer-based ARMS technique is the estimated length of the amplification product. According to previous reports, the length of the cell-free fetal DNA was mostly less than 300 bp, with a median size of 146 bp (Lo et al., 2010). Therefore, based on the previously reported median size of cffDNA, markers with amplicon size less than 300 bp will be preferable. In this study, all primers were designed according to the principle of an amplicon size less than 300 bp. In addition, to distinguish amplification products of similar length, different fluorescent labels are added to primers with the same or a close product length to achieve the detection of multiple mutations simultaneously. Multiplex amplification can greatly reduce the cost of reagents and improve the detection efficiency.

Ramezanzadeh et al. used allele-specific real-time PCR to detect paternally inherited alleles of cffDNA (Ramezanzadeh et al., 2016). However, in their study, DNA size separation was required to enrich fetal DNA. Otherwise, direct detection of cell-free DNA extracted from the maternal plasma by allele-specific real-time PCR would fail. Yang et al. successfully used the ARMS-PCR technique combined with agarose gel electrophoresis to detect the fetal DNA from the amniotic fluid for prenatal diagnosis of choroideremia (Yang et al., 2018). However, the sample they used to detect mutations by the ARMS-PCR method was the fetal DNA extracted from the amniotic fluid and not the cell-free DNA extracted from the maternal plasma. Gisomi et al. identified beta-thalassemia mutations by detecting cell-free DNA in maternal plasma by the RHDO method. Then, fetal DNA extracted from the amniotic fluid or chorionic villus sampling was tested by the ARMS-PCR technique for validation (Mirzaei Gisomi et al., 2020). The RHDO method can be used to detect paternal or maternal variants, but the selection of single-nucleotide polymorphism loci associated with mutant or normal alleles and subsequent Sanger sequencing experiments are relatively cumbersome. In this study, we directly detected mutations of fetal DNA extracted from maternal plasma based on the ARMS-PCR technique combined with CE platforms. Compared with the aforementioned methods, this method is fast, economical, and easy to handle.

In this study, we selected four paternal mutations that could be passed on to the fetus as our target loci. As an exploratory study to explore if the ARMS-PCR technique can be used to detect paternally inherited or *de novo* variants, our results demonstrated its feasibility. However, limited samples and loci were tested in this study. Ideally, more paternally inherited or *de novo* variants should be tested to demonstrate its feasibility. The lack of samples is the biggest shortcoming of this study. In our future work, pathogenic variants that can cause monogenic diseases may be our preferred candidate loci. Supplementary Table S2 lists partial autosomal dominant and autosomal recessive genes that can be tested through the ARMS-PCR technique. For example, achondroplasia is the most common cause of disproportionate short stature (Pauli, 2019). Pathogenic or likely pathogenic variants of the *FGFR3* gene can interpret most achondroplasia. Among them, the pathogenic variant c.1138G>A (p.Gly380Arg) of *FGFR3* was identified in approximately 98% of patients with achondroplasia, while variant c.1138G>C (p.Gly380Arg) was found in approximately 1% of achondroplasia patients (Legare et al., 1993). For families with a history of achondroplasia, especially if the father is the patient or if prenatal ultrasound suggests that the fetus may have achondroplasia, allele-specific primers of c.1138G>A and c.1138G>C can be designed to detect the cell-free fetal DNA from the maternal plasma for prenatal diagnosis purposes through the ARMS-PCR technique.

The ARMS-PCR technique is a promising method for detecting fetal pathogenic mutations, but the method currently has some shortcomings that need improvement. For example, the sensitivity and specificity of some primers are not optimal. Modifying or changing new primers may be required to solve this shortcoming. In addition, limited samples were tested in this study, which is obviously insufficient to verify the screening performance of this technology. Therefore, more samples need to be evaluated before the method can be put into clinical practice.

## 5 Conclusion

In this study, our exploratory study demonstrated the possibility of non-invasive prenatal screening for single-gene disorders through the ARMS-PCR technique. Compared with the NGS method, ARMS-PCR technology is economical, simple, and easy to handle. It may be a promising method for non-invasive prenatal screening for single-gene disorders. However, there is still a long way to go before this technology can be wildly used in clinical practice. In future studies, we will work to develop more primers based on the ARMS technique for currently identified disease-causing variants and to improve the sensitivity and specificity of these primers.

## Data Availability

The original contributions presented in the study are included in the article/Supplementary Material; further inquiries can be directed to the corresponding authors.
