# Supplementary material for: Applying amplification refractory mutation system technique to detecting cell-free fetal DNA for single-gene disorders purpose
Source: Front Genet. 2023 Apr 11;14:1071406. doi: 10.3389/fgene.2023.1071406 (PMC10128035; doi:10.3389/fgene.2023.1071406)
Supplement: Supplementary file 2 [file Table1.DOCX]

Table S1. Primer information

| variants | chromosome location (GRCh37/hg19) | primer ID^a^ | primer sequences^b^ | annealing temperature | amplicon size (bp) |
| --- | --- | --- | --- | --- | --- |
| GJB2  c.235delG | chr13:20763486 | R1 | AGAAGATGGATTGGGGCACG | - | - |
|  |  | F1-A | actACACGAAGATCAGCTGCAGG**G** | 58℃ | 261 |
|  |  | F2-B | ACACGAAGATCAGCTGCAGG**_∧_**CT | 58℃ | 258 |
| DYSF  c.4585C>T | chr2:71883367 | R2 | GACGTCTTCGAGGATTTACCTT | - | - |
|  |  | F2-A | actTAACACCTTCAAGCTGTAC**C** | 58℃ | 95 |
|  |  | F2-B | TAACACCTTCAAGCTGTAG**T** | 58℃ | 92 |
| SLC26A4  c.2236-25T>A | chr7:107352959 | R3 | CAGATGAGAAGCACCAGGAA | - | - |
|  |  | F3-A | actAAAAAGAGAACACAGGGTAG**A** | 58℃ | 95 |
|  |  | F3-B | AAAAAGAGAACACAGGGAAG**T** | 58℃ | 92 |
| PAH  c.158G>A | chr12:103306579 | R4 | CTTGCTTTGTCCATGGAGGT | - | - |
|  |  | F4-A | actAGCACTGACCTCAAATAAG**C** | 58℃ | 202 |
|  |  | F4-B | AGCACTGACCTCAAATAAT**T** | 58℃ | 199 |

^a^Primer name: R denotes the shared reverse primer; F-A: allele specific forward primer for wild-type allele; F-B: allele specific forward primer for mutant-type allele; ^b^ For primer sequences column, the bold letters and “**_∧_**” represent where the target bases are, while the underlined capital letters are deliberate mismatch bases.


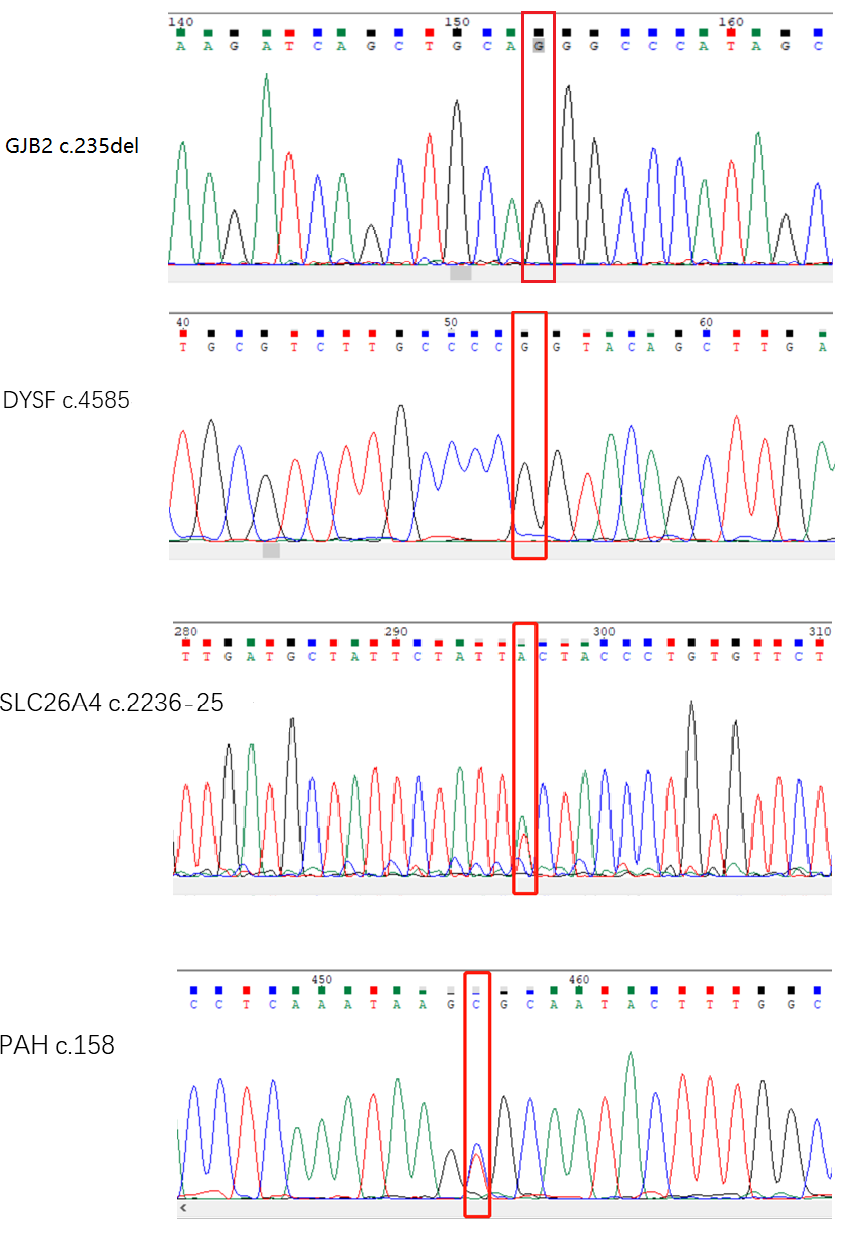


Supplementary figure 1. Sanger sequencing results of genomic DNA extracted from amniotic fluid.
